# Conservative Treatment of the Pulp

**Published:** 1878-09

**Authors:** Walter H. Fundenberg

**Affiliations:** Pittsburg, Pa.


### ARTICLE III.

### *Extracts from a Prize Essay on the "Conservative Treatment of the Pulp."*

BY WALTER H. FUNDENBERG, PITTSBURG, PA.

Presented to the Faculty of the Philadelphia Dental College, for the Degree of D. D. S.

* * * * Every tissue of the human body is liable to pathological changes, and it is therefore not to be supposed that the highly organized nervous expansion which we term "the pulp" is exempt from the operation of this law. Our every day practice shows us that this substance is liable to varying degrees of disease, from simple irritation consequent upon exposure and other causes, to the severest forms of inflammation—these degrees producing according to their intensity, simple hyperæmia—chronic inflammation with resulting textural changes, and intense inflammation, suppuration and death.

The various forms of pulp disease are produced either by external or internal causes. The external are caused by chemical or mechanical influences, the internal by abnormal growths or pulp nodules, and by other obscure changes not yet fully recognized by pathologists. The greater number arise from the external causes, but it cannot be doubted that the internal causes of pulp disease operate much more frequently than is generally supposed. The diagnosis of these cases arising from internal causes is often difficult, but is generally arrived at with a reasonable degree of certainty by a careful review of the symptoms. For instance, take a case in which the patient presents himself with a "toothache"—you make a thorough examination, find the teeth free from apparent disease, with no indication of periosteal trouble—the pain not increased by extremes of heat or cold, and no evidence of a reflex neuralgia. In such a case you will be justified in suspecting osseous deposits in the pulp cavity or at the termination of the root of the tooth, which by pressure establishes either a simple irritation in the tooth or a more extensive neuralgia along the branches of the fifth pair.

In conditions so obscure and so removed from the influence of topical remedies, we will often be unable to afford permanent relief, without the destruction of the pulp.

In the consideration of the affections of the pulp arising from external causes, we find three factors—first those arising from attrition, second, those from mechanical violence, and lastly those from chemical causes. In those caused by attrition we find nature ever conservative in her efforts, throwing out a deposit of secondary dentine as a barrier, shielding the sensitive pulp from the contact of external irritants. Often as the process of attrition goes on, the pulp cavity becomes nearly obliterated without the production of any pain, but in other cases this process is accompanied with high irritation and sometimes suppuration. Fortunately this process occurs mostly in persons of advanced age. The oral teeth are generally the seat of this attrition, usually the six superior, caused by the inferior closing upon their cutting surfaces.

We come lastly to those exposures of the pulp, caused by chemical action. Here we find the tooth structure rapidly breaking down and the pulp doomed to certain destruction if we as conservative practitioners do not step forward and lend a helping hand to nature, and assist her in forming those barriers of defence and protection which she has been endeavoring herself to erect. *Sequare Naturam*, was the golden maxim of the ancients,—follow nature; let her methods of repair and protection serve as hints to yours. In thus endeavouring to define these barriers against the destructive influences that destroy the pulps, we find that many different methods and materials have been used by different practitioners. In looking over the long list, we find that there is no remedy that will not fail occasionally in this department of medicine as in all others. The principal materials used have been the Oxy-chloride of Zinc, Gutta-percha or Hill's Stopping, Metal Caps, Phosphate of Lime, Court Plaster, Isinglass, Gold-beaters Skin, and Egg Membrane; of these, I place the Oxy-chloride of Zinc at the head of the list, as I discard the rest as of little or no value in direct exposure. But in some cases in which it is claimed that this material fails, some of the above may be used, especially in those in which the pulp is *almost* or *very slightly* exposed. Upon deciding to operate, we must carefully remove all irritants, as the pulp will not tolerate foreign substances; we next control the circulation and reduce the inflammation if any exist. This is very important as all exposed pulps die by what may be termed strangulation; if we can succeed in this, we start with a solid foundation. It is claimed by some, that it is impossible to diagnose "inflammation of the pulp." It must be admitted that in some cases it is very difficult; cases that are congested and inflamed, whether recent or of long standing will bleed *freely* if pricked with an instrument. This is a valuable diagnostic mark; in many cases pain due to fullness of the parts, and consequent pressure on the nerve fibers, will assist the diagnosis. In large exposures this symptom may not exist, because there is more room for expansion. With a little careful investigation, errors of diagnosis as to the presence or absence of inflammation or congestion, need not occur.

In cases highly inflamed and congested, oil of cloves, creasote and aconite are useful. The aconite decreases the circulation in the part by constricting the capillary vessels, acting doubtless on the vaso-motor nerves. It is necessary however to be careful in its use, as the vitality of the pulp may be so reduced that its death may result. I find a very useful formula, as used by a prominent practitioner, to be Creasote, gtt. V.— Tr. Iodine and Aconite, aa, ʒ ss, M. This acts with very satisfactory results. In many cases the oil of cloves alone answers admirably, and is preferable because its taste and odor are more pleasant. There are cases however in which we can bring about a condition suitable for capping by the use of creasote or carbolic acid; these remedies act not only specifically, but also by disinfecting the vitiated secretions.

Having now succeeded in preparing the pulp for the reception of a barrier, we now proceed to erect it. What material shall we use? In the list of materials, I have mentioned metal caps, which are principally lead and gold. These are made from a thin sheet of metal of such size and shape as to cover the pulp completely, without coming into contact with it, to accomplish which it is necessary to make the side next to the pulp concave, and also to fit the edges in a groove made in the dentine, thus retaining it *in situ*; of these two metals, lead is certainly more suitable, being more readily adapted, and also less of a conductor. Some operators have arched the gold in filling from each side towards the center; there is no advantage in this and it is besides a difficult operation. But the space that is left between the cap and pulp by these operations constitutes a formidable objection to their use. It is claimed that an osseous deposit will be thrown out here, but I think it much more probable that a fluid exudation will ensue, the result of which will be pressure, pain, inflammation and gangrene. To prevent this occurrence, it has been suggested to place in this space, collodeon or gutta-percha dissolved in chloroform. This may be done if the exposure be very slight, *but only in such cases.* The phosphate of lime mixed with lacto-phosphate has its advocates; this is made into a thick paste, placed in direct contact with the pulp, the cavity sealed and allowed to remain for a time. It is claimed that when this is cut away properly, a perfect capping over the pulp will be left. Egg-membrane, gold-beaters skin, Isinglass, and gutta-percha are all placed over the exposure and the filling inserted. Of the above, none equal the oxychloride of zinc; it is readily applied and appears to be more acceptable to the pulp, not acting so much as a foreign body, if applied correctly, although it can be used in such a manner as to be the most objectionable, causing intense suffering to the patient; but care being taken, we find it will cause sufficient stimulation to excite the pulp to healthy action. Dr. Garrettson, in his valuable work on "Oral Surgery," says in regard to the use of this material for capping, "That oxy-chloride of zinc is a most admirable agent in such direction, employed with a judicious care, is certainly not to be denied." No substance introduced into a tooth seems to exert greater influence in the excitation of that action which produces secondary dentine, than does it; but injudiciously employed, no compound more quickly provokes antagonistic inflammatory action.

The manner of applying this material is this: After being satisfied that the pulp is in as good condition as it is possible to bring it, and that the cavity is perfectly dry, a small portion of the oxide is mixed with creasote on some smooth surface as a piece of glass, to such a consistency that it can be held on the point of an instrument, and placed in direct contact with and over the pulp. The paste thus formed will remain in a soft condition, and lying in close opposition to the pulp, will prevent any effusions taking place, as invariably happens when the covering is arched so as to leave a cavity beneath. It is very often advisable to allow a considerable portion of this material to extend over the cavity, and at the same time of such a thickness as will prevent the next application which is to be a mixture of the oxide and chloride, from giving undue pain. If there is found to be a surplus of creasote in the cavity, by the use of a piece of spunk or any absorbent, we can remove it entirely if desired.

Having placed the oxide and creasote in the cavity, our next step is to remove any that may have come in contact with the edges. If we do not take this precaution, we will find as this material never hardens that we will have an imperfect filling. After this we proceed to insert the oxide and chloride. In mixing this, it should be performed as rapidly as possible, for if the material is once allowed to commence crystallizing we lose one of its important properties, as it never becomes as hard again when once disturbed. Many operators ruin their material in this way. I am sorry to say I have seen a number mix it as though they did not know its properties, and am pained to believe there are men in the profession who use materials simply because others have done so, without knowing their properties or endeavoring to discover them. Again we must not allow too much chloride to be mixed with the oxide as we will have intense pain and sometimes extended irritation. I am satisfied many pulps are destroyed in this manner. Many operators object to this material on this account. I think if they will use Agate Cement, a material which has lately been brought before the profession, they will find the pain can be overcome. I would here say in making all applications, of whatever nature they may be to the pulp, we should endeavor to have them as near the temperature of the tooth as is possible. This may appear to many not to be of much importance, but any person knowing the delicate structure he is dealing with, can at once see the value of such a precaution. Having inserted the material, we then allow it to harden. Guided somewhat by our next procedure, if we propose to wait for secondary dentine, we allow the material to be kept perfectly dry from twenty to forty minutes. If we are to insert a filling over it at the same sitting, we must use our judgment, filling as soon as we feel it will bear a filling properly. I will here give you a record kept by my father, Dr. W. F. Fundenberg, of Pittsburg, of pulps treated in this manner, for the last five years; it being more complete and extensive than my own.

The whole number of pulps capped in that time in the permanent teeth was 306, of these 235 were capped and permanently filled at first sitting; of this great number we are aware of but 11 known failures; of the other 71 that were filled temporarily and afterwards filled permanently at another sitting, we find the small number of 3 failures, or a total of 14 failures in the 306 pulps capped. In the temporary teeth no complete record has been kept, but we find the failures more numerous.

These figures to many will seem enormous, especially those practicing in districts where they claim this can not be accomplished. I do not contend that these 14 are all of that 306 that have failed; it is quite probable others have failed that we are not aware of. I do not believe two-thirds have failed as some practitioners claim to be the result of practice of this kind; and I doubt if one eighth have succumbed.

You will all ask, what do you call a failure? I answer where a pulp has once been capped and it is afterwards necessary to *remove* the *filling* or *tooth.* We find from good authority that some parts of this country are more suitable than others for capping, the South and West as a whole being very favorable, the latter in particular. In the East we find a difference of opinion, although this city, Philadelphia, is numbered as unfavorable; upon this point I am unable to speak, not having treated it as thoroughly as I would have liked, as many of the patients attending clinics at the college are of that class whom it is difficult to keep before you.

I think I have clearly shown in the foregoing, that exposed pulps of teeth can be saved, if the operator of the present and future will only lean towards conservative treatment in the proper manner. I am pleased to see the great number who are conservative, and still adhere to that practice. If we fail, we can only wait until science reveals something more successful. I believe we are justified in many cases in giving the patient the benefit of the doubt, and if not successful we can feel that we made an effort to *save*, not *destroy*, that which was placed there for a purpose, by the wisdom of the Creator.

---

